# Cholera outbreak caused by drinking unprotected well water contaminated with faeces from an open storm water drainage: Kampala City, Uganda, January 2019

**DOI:** 10.1186/s12879-021-07011-9

**Published:** 2021-12-27

**Authors:** Daniel Eurien, Bernadette Basuta Mirembe, Angella Musewa, Esther Kisaakye, Benon Kwesiga, Francis Ogole, Daniel Okello Ayen, Daniel Kadobera, Lilian Bulage, Alex Riolexus Ario, Bao-Ping Zhu

**Affiliations:** 1 Uganda Public Health Fellowship Program, Kampala, Uganda; 2 Kampala Capital City Authority, Kampala, Uganda; 3 Central Public Health Laboratories, Ministry of Health, Kampala, Uganda; 4 US Centers for Disease Control and Prevention, Kampala, Uganda

**Keywords:** Cholera, Outbreak, Capital city, Uganda

## Abstract

**Background:**

Kampala city slums, with one million dwellers living in poor sanitary conditions, frequently experience cholera outbreaks. On 6 January 2019, Rubaga Division notified the Uganda Ministry of Health of a suspected cholera outbreak in Sembule village. We investigated to identify the source and mode of transmission, and recommended evidence-based interventions.

**Methods:**

We defined a suspected case as onset of profuse, painless, acute watery diarrhoea in a Kampala City resident (≥ 2 years) from 28 December 2018 to 11 February 2019. A confirmed case was a suspected case with *Vibrio cholerae* identified from the patient’s stool specimen by culture. We found cases by record review and active community case-finding. We conducted a case–control study in Sembule village, the epi-center of this outbreak, to compare exposures between confirmed case-persons and asymptomatic controls, individually matched by age group. We overlaid rainfall data with the epidemic curve to identify temporal patterns between rain and illnesses. We conducted an environmental assessment, interviewed village local council members, and tested water samples from randomly-selected households and water sources using culture and PCR to identify *V. cholerae*.

**Results:**

We identified 50 suspected case-patients, with three deaths (case-fatality rate: 6.0%). Of 45 case-patients with stool samples tested, 22 were confirmed positive for *V. cholerae* O1, serotype *Ogawa*. All age groups were affected; persons aged 5–14 years had the highest attack rate (AR) (8.2/100,000). The epidemic curve showed several point-source outbreaks; cases repeatedly spiked immediately following rainfall. Sembule village had a token-operated water tap, which had broken down 1 month before the outbreak, forcing residents to obtain water from one of three wells (Wells A, B, C) or a public tap. Environmental assessment showed that residents emptied their feces into a drainage channel connected to Well C. Drinking water from Well C was associated with illness (OR_M–H_ = 21, 95% CI 4.6–93). Drinking water from a public tap (OR_M–H_ = 0.07, 95% CI 0.014–0.304) was protective. Water from a container in one of eight households sampled tested positive for *V. cholerae*; water from Well C had coliform counts ˃ 900/100 ml.

**Conclusions:**

Drinking contaminated water from an unprotected well was associated with this cholera outbreak. We recommended emergency chlorination of drinking water, fixing the broken token tap, and closure of Well C.

## Background

Cholera is an acute diarrheal infection that is characterized by sudden onset of profuse, painless watery diarrhea and vomiting [1]. It can rapidly lead to severe dehydration and death if left untreated [2]. With timely and proper management, the case-fatality rate should be < 1% [3]. Infection occurs when food or water contaminated with the bacterium *Vibrio cholerae* serogroups O1 and O139 are ingested. Epidemics have been exclusively associated with the toxigenic *V. cholerae* O1 serogroup, which consists of two biotypes: classical and El Tor. Each biotype comprises three serotypes: Inaba, Ogawa, and Hikojima [4].

It is estimated that 1.4–4.3 million cholera cases and 28,000–142,000 cholera-related deaths occur every year globally [2]. Access to clean water and food is associated with reduced disease incidence [2]. In recent years, cholera outbreaks have mainly occurred in developing countries, particularly in poor areas with inadequate access to clean water and poor sanitation in Sub-Saharan Africa [5]. In 2019, the World Health Organization (WHO) categorized Uganda among the 51 “endemic” countries for cholera (those reporting confirmed cases during the last 3 years with evidence of local transmission) [2].

Uganda has several cholera hotspots, most along the western border with the Democratic Republic of Congo, in Karamoja region to the north, and in Kampala city slums [6]. Kampala city, like most cities in developing countries, is experiencing rapid urbanization as well as rapid development of informal peri-urban settlements, or slums. More than 60% of the city’s low-income population reside in these densely-populated slums, which have very low levels of basic services (sanitation, water supply, solid waste collection, and storm water disposal) [7, 8]. Most slums in Kampala are situated in low-lying areas, making them susceptible to frequent flooding during heavy rains. The use of pit latrines, the dominant sanitary facilities in the Kampala slums, results in constant risk of fecal contamination of shallow groundwater, which is frequently used as a source of drinking water [8, 9]. Despite being prohibited since 2000, pit latrines are still constructed along drainage channels, where they can release sewage into open drainage lines [10].

On 6 January 2019, the District Surveillance Officer Rubaga division in Kampala notified the Emergency Operation Center (EOC) of the Ministry of Health (MoH) about a suspected cholera outbreak in Sembule village, a slum in Rubaga division. We investigated to confirm the outbreak, identify the source and mode of transmission, and recommended evidence-based interventions to stop the outbreak and prevent future outbreaks.

## Methods

### Outbreak area

**Fig. 1:**
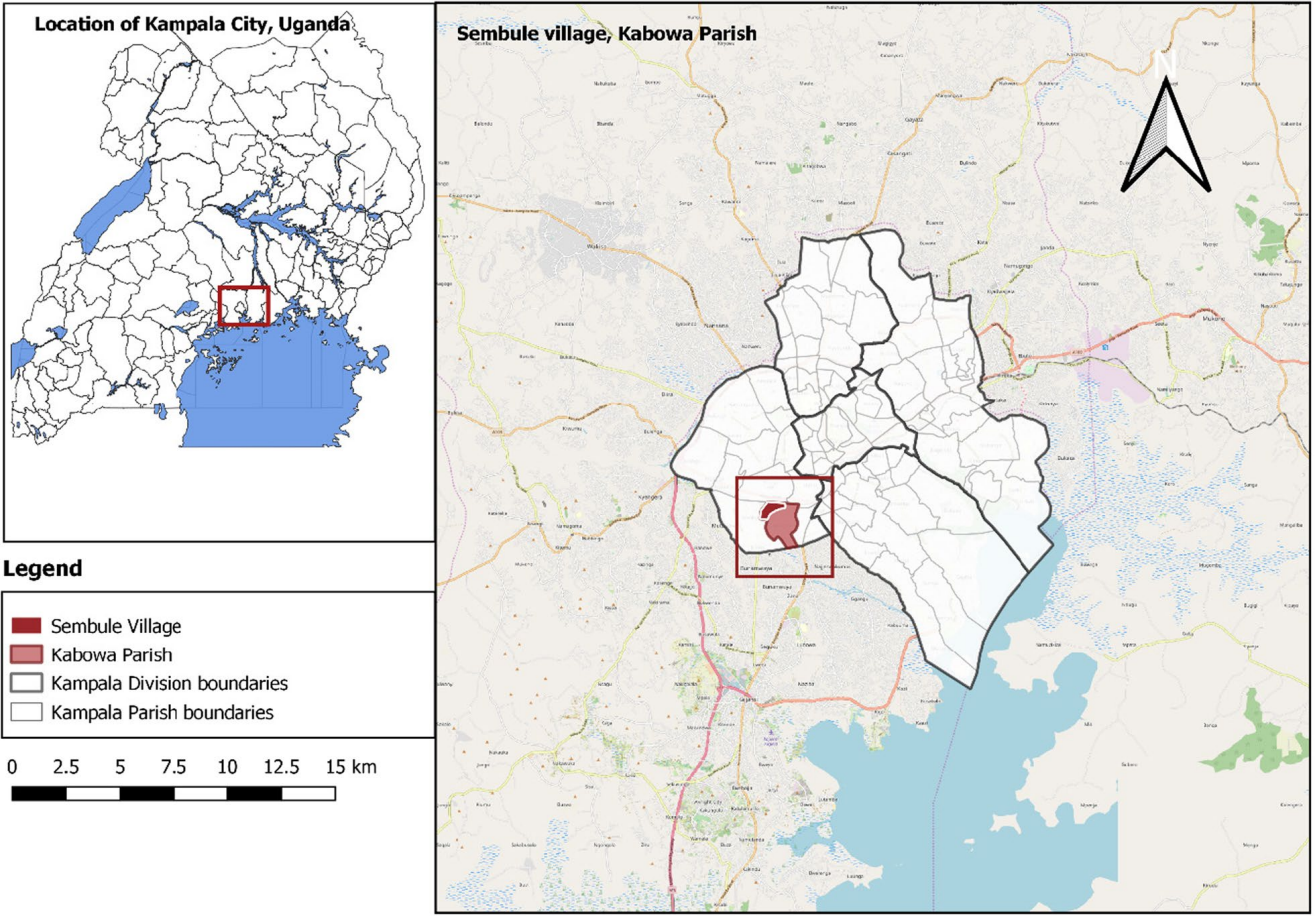
Location of Sembule Village, Kabowa Parish, Kampala City, Uganda

The outbreak occurred in Kampala (the capital city of Uganda), which has an estimated population of 1.6 million [7, 10]. Kampala has five divisions: Kampala Central, Kawempe, Makindye, Rubaga, and Nakawa. We focused our epidemiologic investigation in Sembule village, in Kabowa parish in Rubaga Division (Fig. 1). The village is located on a swamp and was the epi-center of this outbreak. As of January 2019, the village register listed 780 households and a population of approximately 4700 persons. A large proportion of the community comprises mobile persons who conduct petty trade such as vending vegetables and fruits in Kampala city center. One of the city’s major open drainage channels, Nalukolongo channel, passes through the village. The main sources of drinking water in Sembule village include a public token tap, private taps, and open wells. The public token tap is a pre-paid water meter that dispenses low-cost safe water to households and was associated with a pilot program supported by the National Water and Sewerage Corporation (NWSC) and donors starting in 2015 in Kampala slums. The meters are operated by an electronic key, known as a token that is pre-loaded with credit. Any person can buy a key and refill the token with credit at a cost of 0.01 United States Dollar (USD) per 20-l jerry can. As water is dispensed, the meter deducts credit from the token at the official rate. In this way, consumers deal directly with NWSC and this eliminates middlemen from inflating safe water prices.

### Case definition and case-finding

We defined a suspected case as onset of profuse, painless, acute watery diarrhea in a Kampala City resident (≥ 2 years) from 28 December 2018 to 11 February 2019. A confirmed case was a suspected case with *V. cholerae* identified from the patient’s stool specimen by culture or PCR. We reviewed patient records at Naguru Hospital, Lubaga hospital, and Mulago National Referral Hospital to identify suspected case-patients. These three hospitals were designated by MoH to provide cholera diagnostic testing and treatment free of charge to patients from the affected areas. With help from the community health workers, we actively searched for additional cases in households and private clinics in the affected areas.

### Descriptive epidemiology and hypothesis generation

We performed descriptive analysis on case-patients’ clinical presentations, as well as distribution of the case-patients by age, sex, and place of residence. We used village population data from the Local Council chairperson’s village register as the denominator to calculate attack rates. We used epidemic curves to describe case-patients’ dates of symptom onset. We also calculated the case-fatality rate.

To generate hypotheses on possible exposures associated with illness, we interviewed 23 case-patients from the most affected village. We asked case-patients about the type and source of food they ate and the source of the water they drank during the 5 days before their symptom onset.

### Case–control study

To test the generated hypothesis, we conducted a case–control study in Sembule village, where the first cases were reported. We generated a list of all households with a confirmed case-patient, and selected one from each of the 18 affected households. In households with more than one case, we wrote names of the affected persons on paper lots, shuffled in a bowl and picked one. For each case-patient, we selected five control-persons. A control-person was a resident of Sembule village within the same 5-year age-group as the case-patient and without acute watery diarrhea from 28 December 2018 to the time of the investigation. To select control-persons, we obtained a list of households in the village with eligible control-persons, and wrote the names of the household heads on paper lots. We randomly drew out five paper lots from the basket, and attempted to identify an eligible control-person from each of the selected households. If enough control-persons were not identified, we repeated the process until we had enough control-persons for each case.

We administered a questionnaire to the eligible case-patients and control-persons to obtain information on their water exposures. Data were collected on the respondents’ water source, means of storage (jerrycan or open container), whether respondents had a water token or not, whether the respondents treated (using chlorine tablets) or boiled their drinking water, and demographic variables (i.e., age, sex, education level). To measure the associations between exposure variables and illness status, we used the Mantel–Haenszel method to estimate odds ratios (OR) and their 95% confidence intervals, accounting for frequency-matching of case-persons and control-persons.

### Laboratory investigations

Stool samples from 45 case-patients admitted at the three Cholera Treatment Centers (CTCs) were screened using the rapid immunochromatographic diagnostic test Crystal VC™ dipstick (Span Diagnostics Ltd., Surat, India). Approximately 4–6 drops of liquid stool were transferred to a test tube using a pipette that was part of the dipstick package. The dipstick was then inserted into the liquid stool, and the results were read after approximately 15 min. We interpreted results following the manufacturer’s instructions. The appearance of two bands on the dipstick, one control and one test, indicated that the stool sample was positive for *V. cholerae*. Th*e appearanc*e of only the control band indicated a negative sample and the non-appearance of the control band indicated a procedural error [11, 12]. We also transported stool samples from the 45 case-patients in Cary-Blair medium to the Central Public Health Laboratory (CPHL) for culture. Upon arrival in the laboratory, samples from the Cary Blair media were streaked out on Thiosulphate-Citrate-Bile-Salts Sucrose Agar (TCBS™; EIKEN Japan), inoculated in alkaline peptone water (APW) at 37 °C for 18–24 h [14]. An aliquot was streaked out on TCBS and the samples in APW and on TCBS were incubated for 12–24 h at 37 °C. If no growth on TCBS was detected after incubation, an aliquot of the sample in APW was streaked out on TCBS and incubated again. If yellow colonies indicative of *V. cholerae* wer*e detected* on TCBS, motility indole ornithine agar (MIO) and triple sugar iron agar (TSI) were inoculated with colonies from TCBS and incubated for 18 h at 37 °C. In addition, colonies from TCBS were sub-cultivated on gelatin agar for later serological confirmation and incubated at 37 °C overnight. If colonies indicative of *V. cholerae* wer*e observed* on TSI and MIO after incubation, colonies from gelatin agar were tested for agglutination reactions with O1 polyvalent, O1 Inaba, O1 Ogawa and O139 antiserum (Beckton Dickinson, USA).

### Environmental investigations

Based on results from our hypothesis-generating interviews and descriptive epidemiology, we suspected that drinking contaminated water might have been associated with this outbreak. We therefore inspected the main sources of drinking water in Sembule village, including the public token tap, private taps, and open wells. We drew map of the village area using Quantum GIS to show the major sources of water and distribution of cholera case-households. We systematically collected water samples from six public token taps, one household water tap, four open wells, and water storage containers in six randomly-selected households in Sembule village using sterile containers. We used both Most Probable Number (MPN) method and PCR methods using Multiplex PCR assay for detection of O1 and O139 serogroup *V. cholerae* and *ctxA* in water sources [13, 14] and both culture and PCR for water stored in households. We obtained rainfall data for Kampala city around the outbreak period from World Weather Online [15] to establish the temporal association of rain with case counts. We verified the rainfall dates by interviewing the Village Local Council leader and community health workers about the days it rained and any resulting flooding.

## Results

### Descriptive epidemiology and hypothesis generation findings

We identified 50 suspected cholera case-patients as of 11 February 2019 with three deaths (case-fatality rate = 6%). Cases presented with watery diarrhea (100%), vomiting (68%), abdominal pain (12%), and fever (5.8%).

**Fig. 2:**
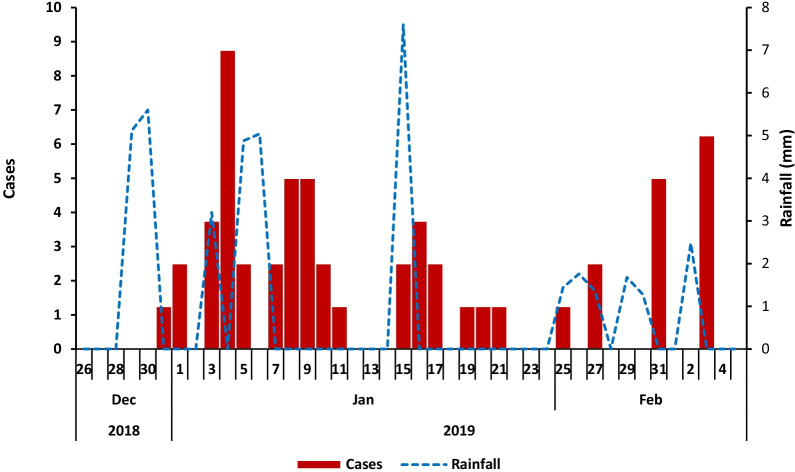
Distribution of symptom onset dates of cholera cases and rainfall: Kampala City, Uganda, December to January 2019

The median age of case-patients was 20 years; with the most affected age-group 5–14 years. Females and males were similarly affected (Table 1). The epidemic curve showed several point-source outbreaks (Fig. 2).

**Table 1:** Attack rates by age-group and sex of cholera case-patients during a cholera outbreak: Kampala City, Uganda, December 2018 to February 2019

Characteristic	Number (n = 50)	Population	Attack rate/100,000
*Age-group*			
< 5	2	225,140	0.88
5–14	25	304,540	8.2
15–24	14	459,630	3
≥ 25	9	621,190	1.4
*Sex*			
Male	24	762,700	3.1
Female	26	862,700	3

**Fig. 3:**
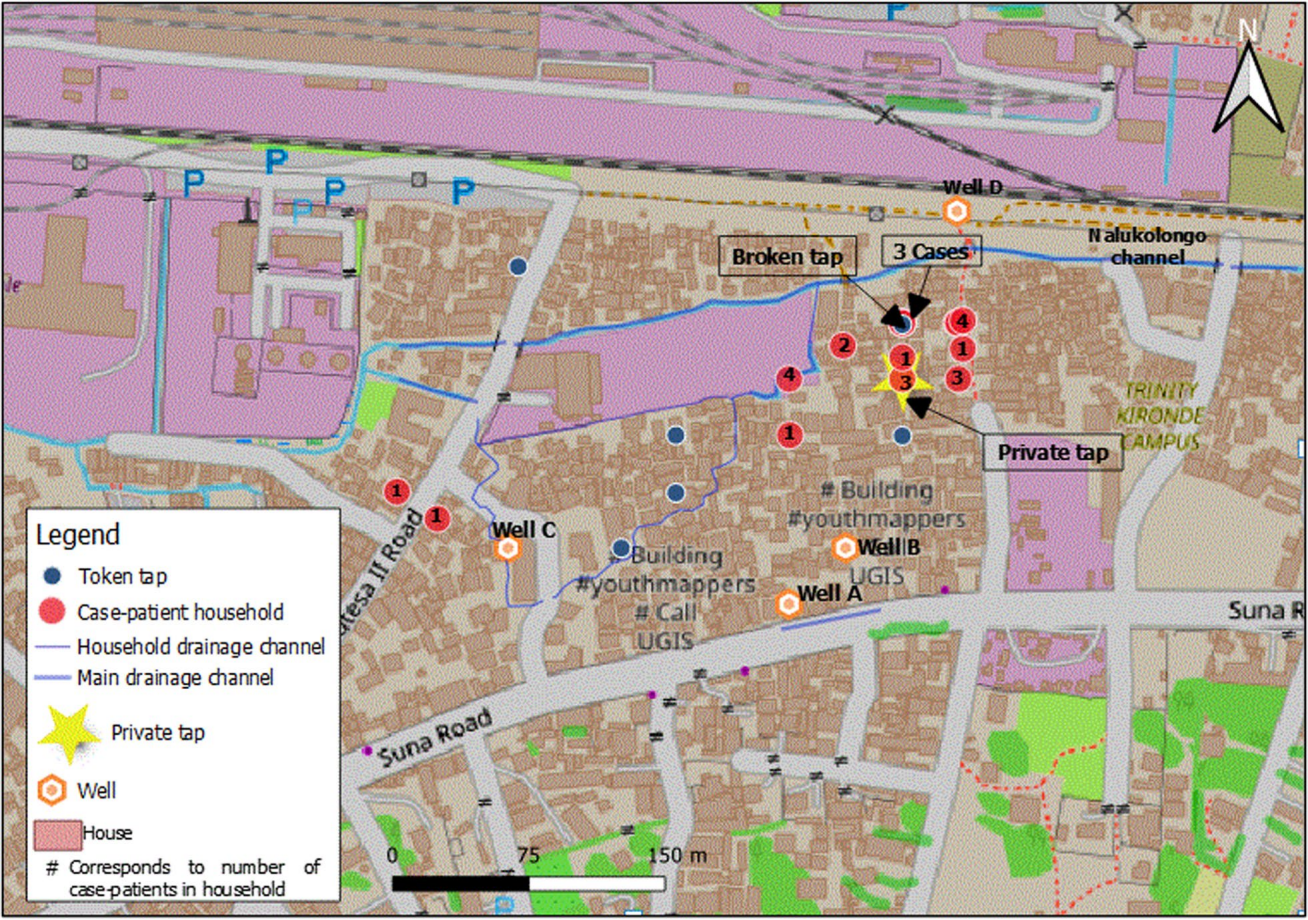
Map showing location of affected households affected by cholera in Sembule village, Kampala, January 2019

All five divisions of Kampala, including Kampala Central, Kawempe, Makindye, Rubaga, and Nakawa were affected Fig. 1 inset), with Rubaga division being the most affected (AR: 6.5/100,000) followed by Central Division (AR: 2.1/100,000). All affected case-patients in Rubaga division came from Sembule village, where the first cases were reported (Fig. 3).

Meteorological data showed that the peaks of the cholera outbreak were preceded by heavy rainfall (Fig. 2). Following declaration of the outbreak on 10 January 2019, the MoH implemented a number of interventions, including community awareness campaigns and distribution of chlorine to households, the number of cases reduced after subsequent rainfall. There were no new cases following blockage of Well C outlets by the Village Local Council on 3 February 2019.

Case-patients reported obtaining water from 10 sources in total, including one private tap, six token taps, and three wells. Of the 23 case-patients interviewed, 22 (96%) had consumed water from Well C within the 5 days before symptom onset; all 22 reported that they did not boil the water. Well C was an unprotected well with water coming from an underground aquifer and an outlet pipe draining directly into the shallow well. Wells A and B were privately-owned protected wells, and the six token taps and private tap were connected to the NWSC and therefore protected (Fig. 3). No single common food was reported eaten by the case-patients during the same period before onset of symptoms. We therefore hypothesized that drinking unboiled water from unprotected Well C was associated with the cholera outbreak.

### Case–control study findings

We evaluated water sources alone and in combination, for persons who reported using more than one water source, for their association with illness. Drinking water from Well C was associated with illness, while all water sources that did not involve water from Well C were neutral or protective (Table 2).

**Table 2:** Distribution of exposure status among case-patients and control persons during a cholera outbreak in Kampala between December 2018 and January 2019

Exposure	N (%) exposed		aOR_M–H_(95%CI)
	Cases (n = 18)	Controls (n = 90)	
Drank water from Well C only	**15 (83)**	**16 (18)**	**21 (4.6–93)**
Drank water both from token tap &Well C	1 (17)	20 (22)	0.7 (0.18–2.7)
Drank water from both tokens tap & Well B	0 (5.6)	5(5.6)	1.0 (0.12–9.1)
Drank water from Well B only	0 (5.6)	6 (6.7)	0.83 (0.11–6.6)
Drank water from token tap	**2 (11)**	**59 (65)**	**0.07 (0.014–0.304)**
Drank water from Well A only	0 (0)	6 (6.7)	0 (0-undefined)
Drank water from both token tap & Well A	0 (0)	6(7.1)	0 (0-undefined)

Bold indicates statistically significant variables

### Laboratory investigation and environmental assessment findings

Of the 45 stool samples collected from suspected case-patients, 22 were culture-positive for *V. cholerae* O1, biotype El Tor serotype *Ogawa*.

Water testing identified no coliforms (Most Probable Number [MPN] 0/100 ml) in samples from public stand water taps and private taps, which is Uganda’s standard for safe drinking water from treated water entering the distribution systems (0 MPN/100 ml) [16]. Samples collected from Well C had total coliform counts ˃ 900 MPN/100 ml. Water from Well C was above the < 10 MPN/100 ml threshold for un-piped water supplies [16]. Of six stored water samples from six households tested, three (50%) had a total coliform count of ≥ 500 MPN/100 ml. The sample obtained from stored water sample from one household tested positive by both culture and PCR for *V. cholerae* O1, biotype El Tor serotype *Ogawa*.

We established that, of the 23 case-patients in Sembule Village, 20 (87%) had previously accessed water from one public token tap, for which they paid United States Dollar (USD) 0.01 per 20-l jerrycan. However, the tap broke down in December 2018, forcing residents to identify other sources of water. While water from Well C was free, a 20-l jerry can from Wells A and B cost USD 0.08, and the same volume cost USD 0.13 from private household taps.

**Fig. 4:**
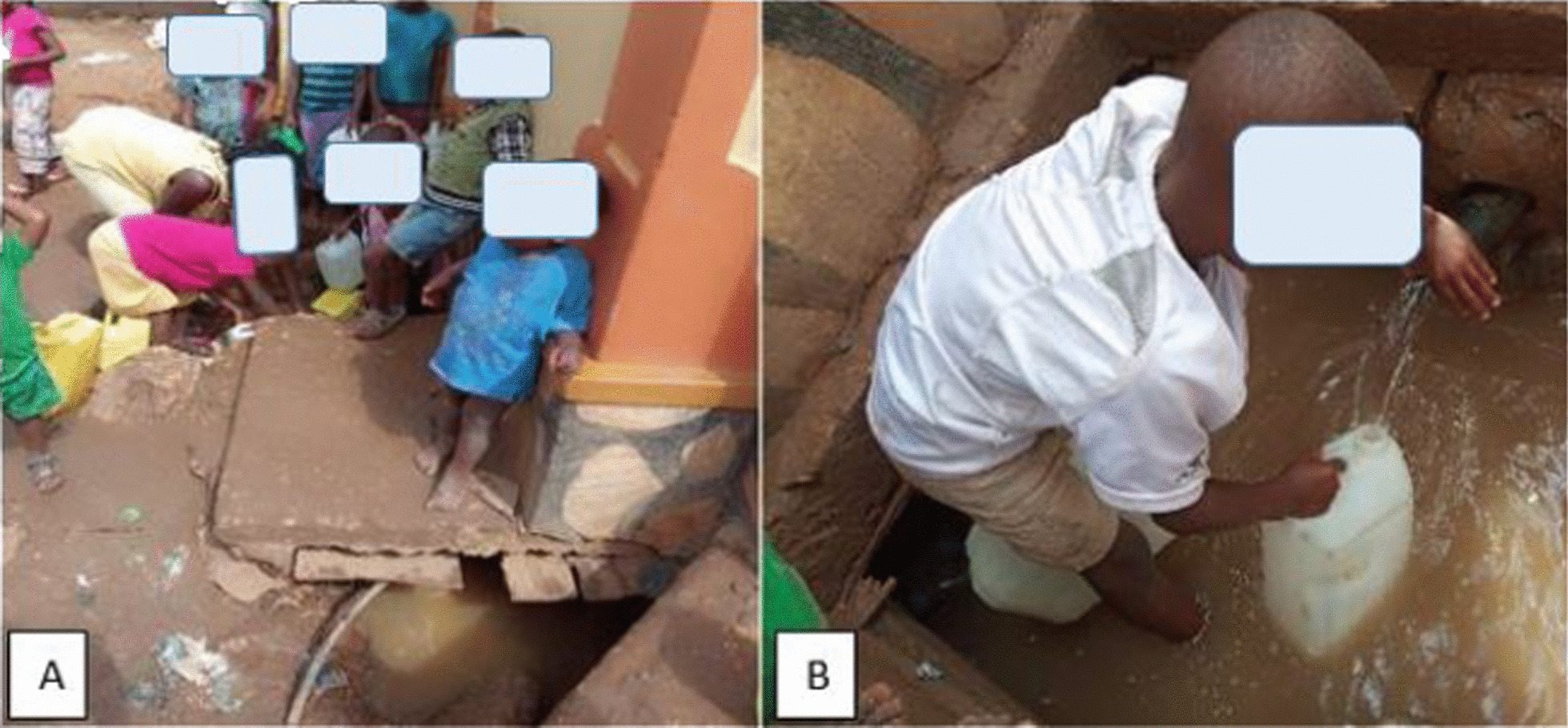
**A** shows children collecting water from well C; **B** shows the outlet pipe of Well C

Well C comprised a small, shallow, unconfined outlet for water coming from a below-ground aquifer. Water flowed into Well C from a pipe fitted perpendicular to the outlet, just above the surface (Fig. 4). The well did not have a permanent cover and the water was flowing continuously into the well from the aquifer. The output pipe from unprotected Well C was approximately 0.3 m above the bottom of an open, informal drainage channel. Most of the community pit latrines were constructed along the drainage channel, to facilitate disposal of waste. Community members often dumped fecal matter into the drainage channel when it rained, to facilitate the washing-away of the waste. Flooding of the drainage channel that resulted from the rain water mixed with the contents of the drainage channel and allowed flow into other nearby areas, including the outlet pipe of unprotected Well C. We found evidence of release of fecal matter into the drainage channel from at least one of the pit latrines constructed along the drainage channel.

## Discussion

Our investigation revealed that a cholera outbreak in Sembule village was associated with drinking unprotected well water. A month before this outbreak, there were reported unconfirmed cholera outbreaks in the neighboring Kisenyi village. Cholera might have been introduced into Sembule village by one or multiple visitors carrying the bacteria, causing the initial contamination when feces were released from their pit latrines into the drainage channel, a common practice in slum areas where latrines are emptied into open drainages during heavy rains. Heavy rainfall likely washed feces in drainage channels into the wells, contaminating the water, which was subsequently consumed without boiling by case-patients in this outbreak.

Globally, poor water and sanitation conditions are often implicated in disease outbreaks, particularly outbreaks of typhoid and cholera [17–20]. These outbreaks are often precipitated or exacerbated by rains [23–25]. Cities in Sub-Saharan Africa are at particularly high risk due to recent population growth without a concomitant increase in access to improved water and sanitation facilities [21, 22]. Previous cholera outbreaks in Kampala city occurred in slum dwellings characterized by overcrowding, insufficient clean water, and poor sanitation facilities [13, 26], and cholera outbreaks in others parts of Uganda have also been associated with drinking untreated water [4, 12–15]. Contamination of the water in such outbreaks has frequently resulted from poor urban planning by city authorities that allowed the development of informal settlements without adequate water and sanitation facilities, and inadequate enforcement of the Public Health Act in slum areas [27].

Many cholera outbreaks in Uganda have not been investigated epidemiologically, and most die out after the implementation of general interventions [28]. The inability to investigate past outbreaks presents a missed opportunity to generate specific evidence-based interventions that, if implemented, might have helped prevent subsequent outbreaks. Results from our investigation provided actionable evidence on the ways well water may be contaminated following release of fecal matter from latrines after rains. We were also able to demonstrate the security of the public piped water system, which remained uncontaminated throughout this outbreak. These data led Kampala Capital City Authority and Ministry of Health to carry out interventions, including distribution of chlorine tablets to household for water disinfection, desilting of the drainage channels to prevent future flooding, treatment of cases, and health education in the communities.

We were unable to identify the initial source of contamination in this outbreak. Identifying the index case can be difficult in cholera outbreaks, as many infected persons are asymptomatic, yet still shed bacteria in their stool [29]. However, definitive identification of the index case in this area might have enabled the provision of more specific recommendations for future prevention.

## Conclusions

In conclusion, this was a cholera outbreak in an urban slum associated with drinking contaminated well water. Fecal contamination of well water likely resulted from the practice of emptying pit latrines into drainage channels during heavy rainfalls. The following public health actions were taken to contain the outbreak: The Ministry of Health distributed chlorine tablets for household water disinfection 9 January 2019, Kampala Capital City Authority emptied pit latrines in Sembule village from 15 to 30 January 2019, the village local council closed Well C on 3 February 2019, and we provided health education to Sembule village residents on prevention of cholera. We recommended treatment and boiling of drinking water. We also recommended increasing the number of public token taps to increase access to safe water, enforcement of chapter 281 of the Public Health Act, and provision of inexpensive latrine-emptying options to dissuade people from releasing fecal matter into open drainages. In addition, to sustainably address the cholera outbreaks in crowded urban settings, Uganda needs to make significant investments in safe water, sanitation and hygiene as prescribed in the *End Cholera*-*Global Roadmap to 2030*.

## Data Availability

The study datasets upon which our findings are based belong to the Uganda Public Health Fellowship Program. For confidentiality reasons, the datasets are not publicly available. However, the data sets can be availed upon reasonable request from the corresponding author and with permission from the Uganda Public Health Fellowship Program.

## Abbreviations

CDC: United States Centers for Disease Control and Prevention
MoH: Ministry of Health
WHO: World Health Organization
CI: Confidence interval
CPHL: Central Public Health Laboratory
OR: Odds ratio
OR_M–H_: Mantel–Haenszel adjusted *OR*s
